# A survey to evaluate the implementation of vaccine recommendations among rheumatologists practicing in Greece

**DOI:** 10.31138/mjr.28.1.41

**Published:** 2017-03-28

**Authors:** Despoina Papadopoulou, Panagiotis Trontzas

**Affiliations:** 1 Pain and Palliative Care Unit, Aretaieio Hospital, National and Kapodistrian University of Athens, Athens, Greece,; 2 Greek Rheumatology Society, Athens, Greece

**Keywords:** Vaccines, Rheumatic diseases, Surveys and Questionnaires

## Abstract

**Objectives:**

We sought to document the knowledge, perceptions and attitudes toward vaccinations among rheumatologists practicing in Greece.

**Methods:**

Rheumatologists practicing in Greece in 2015 were surveyed by questionnaire during the Postgraduate Course of the Greek Rheumatology Society. Statistical analysis was carried out using SPSS software.

**Results:**

One hundred and ten practicing rheumatologists in Greece were surveyed. Response rate was 63%. The majority of responders (82%) inquire patients about vaccination status during rheumatology medical history and most of them specifically advise patients for vaccination uptake (91%). Correct identification of all vaccine types was made by 11% of rheumatologists that took the survey. Ninety-three percent of responders were aware that influenza vaccine should be administered annually, and 94% acknowledged the need for pneumococcal vaccination. Some were not concerned about reduced immunogenicity in patients receiving treatment with methotrexate/TNF inhibitors and rituximab/abatacept (17% and 7%, respectively). A notable percentage overlook that live vaccines are contraindicated during treatment with TNF inhibitors (17%), conventional synthetic DMARDs (61%), or corticosteroids (30%).

**Conclusions:**

According to our results, the majority of Greek rheumatologists have implemented a vaccine strategy in their everyday practice. Still, there are several misconceptions that need to be addressed. A significant percentage cannot properly distinguish between inactivated and live vaccines, and many are not knowledgeable of the potential effect of specific DMARDs on the immunogenicity and safety of vaccination.

## INTRODUCTION

Over the last years, there has been raising awareness of the infection risk among patients with autoimmune rheumatic diseases (ARDs), especially with the use of immune-modulating drugs.^1,2^ National authorities, infectious disease associations and rheumatology societies have developed recommendations in an attempt to increase vaccination coverage, as it is well recognized that a significant burden of infection in ARD-patient population is vaccine preventable.^3,4^

ARD-specific vaccination recommendations are currently lacking in Greece. The National Immunization Committee’s guidelines classify ARD-patients among “immuno-compromised, at-risk populations”.^3^ However, rheumatologic conditions and treatments are not specifically addressed and may not be recognized as “immunosuppressive” by all physicians. An immunization policy was recently developed by the Greek Rheumatology Society (GRS). The purpose of this survey was to document the knowledge, perceptions and attitudes toward vaccinations among rheumatologists practicing in Greece.

## METHODS

The questionnaire was developed in view of the forthcoming immunization policy recommendations by the GRS. Primary goals were to explore rheumatologists’ applied knowledge with respect to immunizations and to record current vaccination practice among Greek rheumatologists. Special interest was focused on influenza, pneumococcal and live vaccines. Future perspective is to track changes in the results after the implementation of the GRS recommendations.

The questionnaires were distributed during the Postgraduate Course of the Greek Society for Rheumatology (10–13 December 2015) to all participants. Inquiries targeted: basic knowledge (e.g., recognition of required and contra-indicated vaccines; identification of vaccine type), availability of recommendations within the department/clinic, specific inquiries as part of medical history, relevant record keeping, and current clinical practice (types and timing of vaccines with regard to treatment). Additionally, rheumatologists were queried about strategies to improve vaccination coverage. The questionnaire is also available online.

All data collected by hard-copy were entered into an Excel worksheet and statistical analysis was performed using SPSS Software.

## RESULTS

One hundred and ten practicing rheumatologists in Greece answered the questionnaires. Response rate was 63% (110/175 course participants). Responders were representative of all rheumatology settings in Greece: 57% worked in private practice, 39% were consultants in hospitals (23%, 10% and 6% in state, university and private hospitals, respectively), and 4% worked in outpatient rheumatology clinics in primary care settings.

### Availability of guidelines

The majority of those affiliated with a hospital or a local or regional health care system (63%, 30/47) answered that they do not have vaccination guidelines in their department. Information about vaccinations is primarily sought in the guidelines of the European League against Rheumatism (41%, 31/97) and systematic literature reviews (41%, 31/97); 30% (23/97) responded that they consult the American College of Rheumatology recommendations, while 20% look up other sources (15/97).

### Applied knowledge about vaccine types, necessity, efficacy and safe administration of vaccination

Correct identification of all vaccine types (**Table 1**) was made by 13 rheumatologists, which corresponds to 15% of rheumatologists that answered this inquiry, and 11% of rheumatologists that took the survey. There was no significant difference in the workplace environment among rheumatologists giving the correct answer to all relevant inquiries (46% in private practice, 54% in hospital settings). Notably, rates of those failing to correctly identify the type of the influenza vaccine, the human papilloma virus vaccine, the hepatitis B virus vaccine and the pneumococcal vaccine were >30%.

**Table 1:** Proper identification of vaccine type

***Vaccine***	***Responders***	***Correct identification (%)***	***Incorrect identification (%)***
**Herpes zoster**	78	68 (88)	10 (12)
**Human Papilloma Virus**	63	28 (44)	35 (56)
**Yellow fever**	60	48 (80)	12 (20)
**Meningococcal**	53	15 (28)	38 (72)
**Influenza**	70	30 (43)	40 (57)
**Pneumococcal**	70	24 (34)	46 (66)
**Hepatitis B**	69	20 (33)	49 (67)

Regarding the two vaccinations most commonly prescribed in patients with ARDs (influenza and pneumococcal vaccines), 93% of responders (86/92) were aware that influenza vaccine should be administered annually, and 94% (87/93) acknowledged the need for pneumococcal vaccination.

When queried about the effect of disease modifying anti-rheumatic drugs (DMARDs) on vaccine-efficacy, 83% (83/100) recognize that the efficacy of both influenza and pneumococcal vaccines may be reduced in patients receiving treatment with methotrexate and/or tumour necrosis factor (TNF) inhibitors; only 5% consider that reduced immunogenicity after vaccination is clinically significant; 17% are not at all concerned about reduced response to vaccinations in patients under methotrexate/anti-TNFs. As for the immunogenicity of influenza and pneumococcal vaccines in patients receiving rituximab or abatacept, 92% (86/93) responded that it may be reduced; 43% recognize that immunity after vaccination in this patient-group may be severely hampered, 46% regard impairment in immunogenicity as minor, while 7% are not concerned about reduced response to vaccinations with these treatments.

With respect to the proper timing to administer live vaccines, many rheumatologists overlooked that they are contraindicated during treatment with TNF inhibitors (17%, 15/89), conventional synthetic DMARDs (61%, 45/74), or corticosteroids administered at doses >20 mg for >2 weeks (30%, 23/75).

### Current practice/attitude toward vaccinations

The majority of responders (82%, 80/97) inquire patients about vaccination status during rheumatology medical history. Amongst them, only 48 answered whether they keep records of patients’ vaccinations; 77% (37/48) responded positively.

The majority answered that they specifically advise patients for vaccination uptake (91%, 100/110). Among those that do not, half (5/10) are sceptical about vaccine safety, 30% (3/10) pointed out limited time, 10% (1/10) question vaccine efficacy and 10% (1/10) do not consider vaccines an important issue in their practice. In regard to specific vaccines, 87% (86/99) responded that they annually prescribe influenza vaccines; 9% (9/99) recommend anti-influenza vaccination but not on an annual basis, whilst 4% (4/99) do not recommend influenza vaccine administration to their patients. Ninety-six % (100/104) recommend vaccination against pneumococcus. Amongst them, 23% recommend the 23valent polysaccharide pneumococcal vaccine (23PPSV), 12% the 13valent conjugate pneumococcal vaccine (13PCV), and 65% administer both vaccines.

Regarding live vaccines, 16% (12/76) did not recognize the need to hold treatment with TNF inhibitors/rituximab for at least 5 drug half-lives before immunization with live vaccines, while 14% (13/90) stated that they do not wait 4 weeks after administration of live vaccine to initiate immunosuppressive treatment.

### Strategies to improve vaccination coverage (Table 2)

**Table 2:** Strategies to improve vaccination coverage

**Strategy**	**Number of responders (%)**
**Shared vaccine records with general practitioners**	69 (73.5)
**Specialized nurses keeping immunization records up to date**	4 (4)
**Vaccine records of patients with ARDs should be created and maintained by rheumatologists**	14 (15)
**Vaccine records of patients with ARDs should be created and maintained by general practitioners**	7 (7.5)

ARDs: autoimmune rheumatic diseases

The majority of Greek rheumatologists (83/87, 95%) consider that the responsibility for vaccination uptake lies with treating physicians and agree that vaccine records of patients with ARDs should be shared between general practitioners and rheumatologists.

## DISCUSSION

The survey results indicate that the majority of Greek rheumatologists have implemented a vaccine strategy in their everyday practice. It is encouraging that per the answers given, most rheumatologists inquire about vaccination history and advise patients to vaccinate. The majority also prescribes influenza/pneumococcal vaccines, following international guidelines.^5,6^ Documentation that the above results are true, however, would require cross-checking with patient chart reviews. An older questionnaire survey among patients demonstrated a suboptimal vaccination rate in Greek patients with rheumatoid arthritis; however, this was prior to the development of EULAR vaccination recommendations.^7^

Notably, almost 8 out 10 recognize that conjugate vaccines may be preferable to polysaccharide or should be given in conjunction with polysaccharide in immunosup-pressed patients.^8^ Another important finding is that there was no difference in the workplace environment in the distribution of answers; this also includes inquiries where identification of the correct answer was requested. The results of a similar survey conducted among Irish rheumatologists were that their practice with regard to vaccination was suboptimal: Most neither recommended nor recorded vaccination history, with the majority feeling that the rheumatology clinic is not the appropriate setting to target strategies to improve vaccine compliance.^9^

Nevertheless, the results of the survey demonstrate that despite the interest that Greek rheumatologists have in vaccines, there are several misconceptions that need to be addressed. A significant percentage cannot properly distinguish between inactivated and live vaccines. That means that they may not properly identify the specific rules which apply for each vaccine category. Consequently, there may be potential risks for patient safety and vaccine efficacy. Additionally, many are not knowledgeable of the potential effect of specific DMARDS on the immunogenicity and safety of vaccination. International guidelines clearly state that live vaccines should be avoided during immunosuppressive therapy, as their administration bears the potential risk of invasive infection with the attenuated vaccine strain.^10^ Unfortunately, this is not recognized by a considerable percentage of Greek rheumatologists, who either do not interrupt treatment at all, or do not withhold therapy long enough before administering a live vaccine. Among live vaccines, the herpes zoster virus (HZV) vaccine can be administered during treatment with csDMARDs, according to the most recent ACR Guidelines for rheumatoid arthritis;^6^ however, standard of care with regard to HZV was not specifically queried during the survey. When it comes to steroids, the answers given indicate that some rheumatologists may not be aware of the steroid dose threshold that impairs the activity of the immune system. The immunosuppressive effects of steroid treatment vary, but a dose equivalent to a total of 20 mg/day of prednisone is considered sufficiently immunosuppressive to raise concern about the safety of immunization with live-virus vaccines. However, there is no consensus as to what is a low dose of steroid; 10mg per day or below is thought to be a sensible compromise. According to the Advisory Committee on Immunization Practices recommendations, vaccination with live vaccines should be deferred for at least 1 month after discontinuation of therapy before administering a live-virus vaccine to patients who have received high-dose, systemic steroids for greater than or equal to 2 weeks.^11^

The immunogenicity of inactivated vaccines is generally preserved during treatment with conventional DMARDs and anti-TNF drugs, though there have been several reports of diminished response to vaccination in patients under methotrexate and/or anti-TNF regimens.^2,12,13^ Most importantly, immune response to influenza and/or pneumococcal vaccination may be hampered as a result of treatment with abatacept and rituximab.^2,14,15^ This explains why guidelines advise to vaccinate prior to the initiation, or after adequate cessation (at least 2 weeks) of immunomodulators, especially when it comes to anti-CD20 and CTLA4 analog treatment,^3,5^ although data about abatacept are conflicting.^16^ However, it appears that many rheumatologists in Greece do not take into account this important aspect regarding the proper time to administer inactivated vaccines with regard to immunosuppressive therapy.

Another noteworthy finding is that a non-negligible percentage of rheumatologists remain very cautious regarding vaccine safety, or question vaccine efficacy in patients with ARDs and prefer not to vaccinate patients. Another small but not unimportant group does not consider vaccines as an essential aspect of rheumatology practice that would reduce infection burden. Additionally, among those that have taken up vaccines in their current practice, some have misinterpretations of guidelines; a characteristic example is that almost 1 out of 10 vaccinates patients against influenza sporadically, but not on an annual basis.

## STRENGTHS AND WEAKNESSES

A limitation of the survey that we would like to acknowledge is that that some respondents filled up the questionnaire form poorly. It is also possible that some may have misinterpreted a question, or may have manipulated their answers. To overcome this weakness, we ensured that the survey was anonymous and responders had sufficient time to consider the questions before answering. The study has important strengths: to our knowledge, this is the first survey in Greece and the second worldwide to document the knowledge, attitudes and perceptions of practicing rheumatologists about vaccinations.^9^ The number of responders is very satisfactory, and the sample is representative of all rheumatology settings in Greece. Most importantly, the survey can be used as baseline for later survey, to compare results and evaluate changes following the development of GSR vaccination recommendations in patients with ARDs.

## CONCLUSION

There are many encouraging findings in this survey that reflect the interest of most Greek rheumatologists in vaccinations and their intention to implement international recommendations. Still, some remain skeptical toward vaccines, some do not possess basic immunization knowledge, and others have misconceptions in their vaccination practice. The GRS guidelines may serve toward increasing vaccine awareness, clarifying misperceptions and assisting in the direction of safe and efficient vaccine uptake.

## Figures and Tables

**Table:** 

**Physician Affiliation/Institution:**	RESULTS:
University Hospital	12
Hospital NHS	27
Private hospital	7
First-tier National Health Network	5

Private Practice	66

**Table:** 

**Is there availability of written instructions for patient vaccines at your workplace: YES/NO**	**YES**	30	**NO**	67
If not, where do you look for information on the vaccination of patients:	RESULTS:			

American College of Rheumatology	30			
European League against Rheumatism	41			
Literature review	41			
Other	20			
Don’t look for information				

**Table:** 

**Do you discuss the need for vaccination with your patients? YES/NO**	**YES**	100	**NO**	10
**If NO, choose the main reason:**	RESULTS:			

Insufficient time	3			
I do not think that it is major issue	1			
I am skeptical regarding the safety of vaccines	5			
I question the effectiveness of vaccines	1			

**Table:** 

**Do you believe the responsibility for ensuring compliance to vaccination instructions belongs to health professionals? YES/NO**	**YES**	83	**NO**	4
**Choose an action plan that will help increase vaccination coverage in patients with ARDs: (chosse 1 reply)**	RESULTS:			

Common patient care by rheumatologists/general practitioners using a ‘vaccination passport’	69			
Specialized nurses dealing with vaccines	4			
Vaccination is primarily the responsibility of rheumatologists	14			
Vaccination is primarily the responsibility of general practitioners	7			

**Table:** 

**Do you include questions about vaccination history in your clinical practice? YES NO**	**YES**	80	**NO**	17
If YES, do you record the answers? YES/NO	**YES**	37	**NO**	11
Herpes zoster	**YES**	69	**NO**	9
Human Papilloma Virus	**YES**	28	**NO**	35
Yellow fever	**YES**	48	**NO**	12
Meningococcal	**YES**	15	**NO**	38
Influenza	**YES**	30	**NO**	40
Pneumococcal	**YES**	24	**NO**	45
Hepatitis B	**YES**	23	**NO**	46

**Table:** 

**Administering live vaccines is contraindicated in patients who receive treatment with:**				
anti-TNF	**YES**	74	**NO**	15
csDMARDs	**YES**	29	**NO**	45
corticosteroids at any dose	**YES**	10	**NO**	56
corticosteroids at a dose of >20mg for >2 weeks	**YES**	52	**NO**	23

**Table:** 

**Live vaccines should be given 1 month before commencement of conventional/biological DMARDs. YES/NO**	**YES**	77	**NO**	13

**Live vaccines should be administered 5 T ½ after discontinuation of anti-TNF and 6–12 months after discontinuation of rituximab. YES/NO**	**YES**	64	**NO**	12

**Annual vaccination against influenza is recommended in patients with autoimmune rheumatic diseases. YES/NO**	**YES**	86	**NO**	6

**Vaccination against pneumoccocus is recommended in patients with autoimmune rheumatic diseases. YES/NO**	**YES**	87	**NO**	6

**Table:** 

**Do you recommend vaccination against influenza to your patients?**	RESULTS:
Yes, every year	86
Yes, but not every year	9
No	4

**Table:** 

**Do you recommend vaccination against pneumococcus to your patients? YES/NO**	**YES**	100	**NO**	4
**If so, which vaccine do you recommend?**	RESULTS:			

23valent polysaccharide	23			
13valent conjugate	12			
Combination of both	65			

**Table:** 

**Patients treated with methotrexate and anti-TNFs may have a reduced immune response after a vaccination against influenza/pneumococcus.**	RESULTS:
Yes, but the possible reduction is small/average	78
Yes, significant reduction	5
No	17

**Table:** 

**Patients treated with rituximab or abatacept may have a reduced immune response after a vaccination against influenza/pneumococcus.**	RESULTS:
Yes, but the possible reduction is small/average	46
Yes, significant reduction	40
No	7

## References

[1] Westra J Rondaan C van Assen S Bijl M Vaccination of patients with autoimmune inflammatory rheumatic diseases. Nat Rev Rheumatol 2015;11:135–45. 25486980 10.1038/nrrheum.2014.206

[2] Papadopoulou D Tsoulas C Tragiannidis A Sipsas N V Role of vaccinations and prophylaxis in rheumatic diseases. Best Pract Res Clin Rheumatol 2015;29:306–18. 26362746 10.1016/j.berh.2015.02.001

[3] Papadopoulou D Sipsas N V Comparison of national clinical practice guidelines and recommendations on vaccination of adult patients with autoimmune rheumatic diseases. Rheumatol Int 2014;34:151–63. 24322451 10.1007/s00296-013-2907-9

[4] Mandell B F Vaccination: an option not to be ignored. Cleve Clin J Med 2010;77:151. 20200164 10.3949/ccjm/77a/03001

[5] van Assen S Agmon-Levin N Elkayam O Cervera R Doran M F Dougados M EULAR recommendations for vaccination in adult patients with autoimmune inflammatory rheumatic diseases. Ann Rheum Dis 2011;70:414–22. 21131643 10.1136/ard.2010.137216

[6] Singh J A Saag K G Bridges S L Jr Akl E A Bannuru R R Sullivan M C 2015 American College of Rheumatology Guideline for the Treatment of Rheumatoid Arthritis. Arthritis Care Res (Hoboken) 2016;68:1–25. 26545825 10.1002/acr.22783

[7] Koutsogeorgopoulou L Antoniadis C Vassilopoulos D Kassimos D Preventive influenza vaccination for patients with rheumatoid arthritis. A need for an international campaign. Clin Rheumatol 2009;28:103. 18953623 10.1007/s10067-008-1024-8

[8] Bühler S Eperon G Ribi C Kyburzd D van Gompele F Visserfet L G Vaccination recommendations for adult patients with autoimmune inflammatory rheumatic diseases. Swiss Med Wkly 2015;145:w14159. 26218860 10.4414/smw.2015.14159

[9] McCarthy E M Azeez M A Fitzpatrick F M Donnelly S Knowledge, attitudes, and clinical practice of rheumatologists in vaccination of the at-risk rheumatology patient population. J Clin Rheumatol 2012;18:237–41. 22832287 10.1097/RHU.0b013e3182611547

[10] van Assen S Elkayam O Agmon-Levin N Cerverae R Doranf M F Dougados M Vaccination in adult patients with auto-immune inflammatory rheumatic diseases: a systematic literature review for the European League Against Rheumatism evidence-based recommendations for vaccination in adult patients with auto-immune inflammatory rheumatic diseases. Autoimmun Rev 2011;10:341–52. 21182987 10.1016/j.autrev.2010.12.003

[11] ACIP Adult Immunization Work Group Bridges C Woods LD Coyne-Beasley T Advisory Committee on Immunization Practices (ACIP) Recommended Immunization Schedule for Adults Aged 19 Years and Older — United States, 2013. MMWR 2013;62:9–19. 23364303

[12] Brezinschek H P Hofstaetter T Leeb B F Haindl P Graninger W B Immunization of patients with rheumatoid arthritis with antitumor necrosis factor alpha therapy and methotrexate. Curr Opin Rheumatol 2008;20:295–9. 18388521 10.1097/BOR.0b013e3282ffdeca

[13] Aringer M Vaccination under TNF blockade - less effective, but worthwhile. Arthritis Res Ther 2012;14:117. 22577892 10.1186/ar3808 PMC3446474

[14] Kapetanovic M C Saxne T Jönsson G Truedsson L Geborek P Rituximab and abatacept but not tocilizumab impair antibody response to pneumococcal conjugate vaccine in patients with rheumatoid arthritis. Arthritis Res Ther 2013;15:R171. 24286269 10.1186/ar4358 PMC3978887

[15] Westra J van Assen S Wilting K R Land J Horst G de Haan A Rituximab impairs immunoglobulin (Ig)M and IgG (subclass) responses after influenza vaccination in rheumatoid arthritis patients. Clin Exp Immunol 2014;178:40–7. 24889761 10.1111/cei.12390 PMC4360192

[16] Alten R Bingham C O Cohen S B Curtis J R Kelly S Wong D Antibody response to pneumococcal and influenza vaccination in patients with rheumatoid arthritis receiving abatacept. BMC Musculoskelet Disord 2016;17:231. 27229685 10.1186/s12891-016-1082-z PMC4880815

